# A Severe Dementia Syndrome Caused by Intron Retention and Cryptic Splice Site Activation in *STUB1* and Exacerbated by *TBP* Repeat Expansions

**DOI:** 10.3389/fnmol.2022.878236

**Published:** 2022-04-14

**Authors:** Marlen Colleen Reis, Julia Patrun, Nibal Ackl, Pia Winter, Maximilian Scheifele, Adrian Danek, Dagmar Nolte

**Affiliations:** ^1^Institut für Humangenetik, Justus-Liebig-Universität Giessen, Giessen, Germany; ^2^Psychiatrische Dienste Thurgau, Münsterlingen, Switzerland; ^3^Neurologische Klinik und Poliklinik, Klinikum der Universität München, Munich, Germany; ^4^Institut für Nuklearmedizin, Klinikum der Universität München, Munich, Germany

**Keywords:** dementia syndrome, intron retention, cryptic splice site, *STUB1*, SCA48, *TBP*, SCA17

## Abstract

Heterozygous pathogenic variants in the STIP1 homologous and U-box containing protein 1 (*STUB1*) gene have been identified as causes of autosomal dominant inherited spinocerebellar ataxia type 48 (SCA48). SCA48 is characterized by an ataxic movement disorder that is often, but not always, accompanied by a cognitive affective syndrome. We report a severe early onset dementia syndrome that mimics frontotemporal dementia and is caused by the intronic splice donor variant c.524+1G>A in *STUB1*. Impaired splicing was demonstrated by RNA analysis and in minigene assays of mutated and wild-type constructs of *STUB1*. The most striking consequence of this splicing impairment was retention of intron 3 in *STUB1*, which led to an in-frame insertion of 63 amino acids (aa) (p.Arg175_Glu176ins63) into the highly conserved coiled-coil domain of its encoded protein, C-terminus of HSP70-interacting protein (CHIP). To a lesser extent, activation of two cryptic splice sites in intron 3 was observed. The almost exclusively used one, c.524+86, was not predicted by *in silico* programs. Variant c.524+86 caused a frameshift (p.Arg175fs*93) that resulted in a truncated protein and presumably impairs the C-terminal U-box of CHIP, which normally functions as an E3 ubiquitin ligase. The cryptic splice site c.524+99 was rarely used and led to an in-frame insertion of 33 aa (p.Arg175_Glu176ins33) that resulted in disruption of the coiled-coil domain, as has been previously postulated for complete intron 3 retention. We additionally detected repeat expansions in the range of reduced penetrance in the TATA box-binding protein (*TBP*) gene by excluding other genes associated with dementia syndromes. The repeat expansion was heterozygous in one patient but compound heterozygous in the more severely affected patient. Therefore, we concluded that the observed severe dementia syndrome has a digenic background, making *STUB1* and *TBP* important candidate genes responsible for early onset dementia syndromes.

## Introduction

Severe familial dementia syndromes are devastating and as yet incurable diseases that have variable clinical signs and symptoms and a broad genetic background. Early onset familial Alzheimer’s disease is one such syndrome that is mainly associated with pathogenic variants in the genes *PSEN1*, *PSEN2*, and *APP* (Bird, 1998/2018; Van Cauwenberghe et al., 2016). Clinical and genetic overlap exists with frontotemporal dementia (FTD), which is caused by mutations in *C9orf72*, *MAPT*, *GRN*, *TARDPB*, *VCP*, as well as other genes (Hodges and Piguet, 2018). Familial dementia is also associated with neurodegenerative diseases such as Creutzfeldt-Jakob disease, Huntington’s disease (HD), and some subtypes of spinocerebellar ataxia (SCA). The SCAs are a heterogeneous group of mostly adult-onset neurodegenerative disorders with autosomal dominant inheritance. Clinical features include ataxia of gait and stance, dysarthria, and abnormal eye movements in combination with other cerebellar signs and symptoms. To date, at least 40 different SCA genes have been described (Bird, 1998/2019; Klockgether et al., 2019). Among these, SCA17 (OMIM#607136) is caused by CAG/CAA repeat expansions in the coding sequence of the TATA box-binding protein (*TBP*; OMIM *600075) gene and is associated with cognitive decline, psychiatric features, and HD-like symptoms (Koide et al., 1999; Toyoshima et al., 2005/2019).

Recently, heterozygous variants in the STIP1 homologous and U-box containing protein 1 (*STUB1*; OMIM *607207) gene have been associated with adult-onset autosomal dominant inherited SCA48 (Genis et al., 2018). In SCA48 patients (OMIM#618093), typical symptoms of ataxia are often accompanied by a cerebellar cognitive affective syndrome (Genis et al., 2018; Lieto et al., 2020; Roux et al., 2020). In some cases, cognitive impairment preceded movement disorder (Genis et al., 2018; De Michele et al., 2019; Palvadeau et al., 2020; Roux et al., 2020; Pakdaman et al., 2021; Ravel et al., 2021). Previously, homozygous or compound heterozygous variants in *STUB1* have been described as autosomal recessive SCA type 16 (SCAR16, OMIM#615768), a severe disease type associated with a broad range of symptoms that usually begin in childhood or adolescence (Shi et al., 2013, 2014; Synofzik and Németh, 2018). During the submission of this manuscript, a SCA17/HD-like phenotype was associated with digenic inheritance of *STUB1* variants in combination with intermediate-range *TBP* repeat expansions (Magri et al., 2022).

*STUB1* encodes the C-terminus of HSP70-interacting protein (CHIP), a 35 kDa protein consisting of 303 amino acids (aa) that is involved in protein quality control pathways (Ballinger et al., 1999). CHIP is composed of three N-terminal tetratricopeptide (TRP) repeats of 34 aa each, a middle coiled-coil domain essential for dimerization (Nikolay et al., 2004), and a C-terminal U-box (Ballinger et al., 1999). CHIP acts as a co-chaperone *via* the TRP repeats and interacts with heat shock proteins (HSP) to enable remodeling of misfolded proteins. In contrast, the U-box functions as an E3 ubiquitin ligase that mediates degradation of misfolded proteins through ubiquitylation (Ballinger et al., 1999; Jiang et al., 2001; Kampinga et al., 2003).

Point mutations in *STUB1*, including predominantly missense and nonsense variants, are the molecular cause of SCA48/SCAR16; small deletions causing frameshift have also been described (Shi et al., 2013; Depondt et al., 2014; Heimdal et al., 2014; Synofzik et al., 2014; Bettencourt et al., 2015; Kawarai et al., 2016; Gazulla et al., 2018; Genis et al., 2018; De Michele et al., 2019; Madrigal et al., 2019; Chen et al., 2020, 2021; Chiu et al., 2020; Lieto et al., 2020; Mol et al., 2020; Olszewska and Kinsella, 2020; Palvadeau et al., 2020; Roux et al., 2020; Mengel et al., 2021; Pakdaman et al., 2021; Park et al., 2021; Ravel et al., 2021; Magri et al., 2022). The latter may cause truncation of CHIP and/or degradation of the defective mRNA *via* nonsense-mediated decay. Point mutations affecting splice sites have been detected less frequently. Only a few variants are located in the conserved splice donor or splice acceptor boundaries (Cordoba et al., 2014; Olszewska and Kinsella, 2020; Roux et al., 2020; Saft et al., 2021; Magri et al., 2022). None of these have been studied in terms of interference with the splicing processes. Impaired splicing can result in exon skipping, activation of cryptic splice sites, or, more rarely, retention of an intron (Wang and Burge, 2008; Scotti and Swanson, 2016).

This study was conducted to elucidate the genetic cause of a dementia syndrome that phenotypically mimics FTD and to determine the underlying molecular pathomechanism.

## Patients and Methods

### Patients

Two affected members of a German family with a dementia syndrome were examined and diagnosed at a specialized center for neuropsychiatric disorders. The family history was consistent with autosomal dominant inheritance. The index patient’s legal guardians provided written informed consent for blood sample donation according to the guidelines of the German Genetics Diagnostics Act. The study was performed in accordance with the principles of the Declaration of Helsinki and was approved by the ethics committee of the Justus-Liebig-University of Giessen (AZ24/14erw).

### Genetic Analysis

DNA was extracted from peripheral blood samples using standard procedures. Point mutations in the dementia-associated genes *MAPT*, *GRN*, *TARDBP*, *PSEN1*, *PSEN2*, and *APP* had been excluded previously. Fragment-length analysis at the corresponding loci for FTD/amyotrophic lateral sclerosis (*C9orf72*), HD (*HTT*), and SCA17 (*TBP*) was performed by polymerase chain reaction (PCR) using a specific fluorescent-labeled primer in combination with an unlabeled reverse primer. Primer sequences are shown in Supplementary Table 1A. Fragments were run on a SeqStudio Genetic Analyzer (Applied Biosystems, Austin, TX, United States) and repeat sizes were determined using GeneMapper v.5 software (Applied Biosystems). DNA was analyzed for variants in *STUB1* (ENSG00000103266) by amplification and sequencing of the seven coding exons, the flanking sequence of intron 1, and the complete intronic sequences (IVS 2-6) of transcript ENST00000219548.9. Primer sequences are shown in Supplementary Table 1B.

Subsequently, whole exome sequencing was performed on the DNA of the index patient, examining all coding regions including flanking intronic sequences. SureSelect XT Human All Exon V7 kit (Agilent, Santa Clara, CA, United States) was used for enrichment. Quality control of the prepared library was performed using a Qubit 3.0 fluorometer (ThermoFisher Scientific, Waltham, MA, United States). Sequencing was performed on an Illumina NovaSeq platform (Illumina, San Diego, CA, United States). The mean coverage of exons was at least > 20× with > 98.5% targeted bases covered. GRCh37 was used as the reference genome. Filter criteria for pathogenic variants were minor allele frequency (MAF) < 0.005 in the gnomAD v2.1.1 database (Karczewski et al., 2020) and Combined Annotation Dependent Depletion score ≥ 25 (Kircher et al., 2014; Rentzsch et al., 2021).

### Bioinformatic Prediction

Impact of the observed splice donor variant was analyzed by *in silico* analysis using the programs MutationTaster2 (Schwarz et al., 2014), NetGene2 (Brunak et al., 1991), and Alternative Splice Site Predictor (ASSP) (Wang and Marín, 2006). The detected variant was subsequently classified according to American College of Medical Genetics (ACMG) guidelines (Richards et al., 2015) and the recommendations for canonical splice site variants (Abou Tayoun et al., 2018).

### Expression Studies

Total RNA was extracted from PAX-conserved (PreAnalytiX, Hombrechtikon, Switzerland) whole blood of the index patient and a healthy control using the Monarch total RNA miniprep kit (New England Biolabs, Ipswich, MA, United States). Additional DNase I digestion (Invitrogen, Waltham, MA, United States) was performed to remove traces of genomic DNA. RNA was transcribed into cDNA using random primers and SuperScript IV reverse transcriptase (Invitrogen). Reverse transcription PCR (RT-PCR) was performed using primers STUB1_cEx2F (5’-CGCTGGTGGCCGTGTATTAC-3’), and STUB1_cEx4R (5’-GTGCTTGGCCTCAATGCAGG-3’). To confirm the integrity of the RNA and the transcribed cDNA, a 210 bp fragment was amplified with primers TBP_Ex5F (5’-TTGCTGCGGTAATCATGAGG-3’) and TBP_Ex6R (5’-GAAACTTCACATCACAGCTCCC-3’) derived from the *TBP* gene as an internal control.

Products were separated on 1.5% agarose gels. Different sized RT-PCR fragments were excised from the gel, purified, and either sequenced directly or cloned into vector pCR4-TOPO using the TOPO TA Cloning Kit (ThermoFisher Scientific). At least 25 clones of each fragment were sequenced.

### Minigene Constructs and Splicing Analysis

Two minigenes were produced: one carried the splice donor variant c.524+1G>A (pcDNA3.1-*STUB1*-Mut) and the other corresponded to the wild-type sequence (pcDNA3.1-*STUB1*-WT). Both minigenes contained the genomic sequences of partial intron 1 to intron 4 of *STUB1*. In brief, primers STUB1_IVS1F_*Bam*HI (5’-TATGGATCCGTACTCCACTGTGCACAGATCC-3’) and STUB1_Int4R_*Eco*RI (5’-TATGAATTCCACCCCACCTCCCTC CTCCA-3’) were used to amplify 995 bp fragments from the genomic DNA of the patient and the control. PCR products were restricted with *Bam*HI and *Eco*RI (New England Biolabs), and subsequently cloned in pcDNA3.1+ (ThermoFisher Scientific). Competent OneShot Top-10 *E. coli* cells (ThermoFisher Scientific) were transformed with the recombinant plasmids. Integrity of the selected clones was confirmed by sequencing.

### Transfection

Chinese hamster ovary (CHO)-K1 cells (American Type Culture Collection, Manassas, VA, United States) were transiently transfected with 0.2 μg of either pcDNA3.1-*STUB1*-Mut, pcDNA3.1-*STUB1*-WT, or pcDNA3.1+ using FuGENE (Promega, Madison, WI, United States). Untransfected cells served as a control. Cells were cultured in F-12K medium (ThermoFisher) supplemented with 10% fetal bovine serum (Sigma-Aldrich, St. Louis, MO, United States) and 1% penicillin/streptomycin (Gibco, Waltham, MA, United States). Cells were harvested after 28 h of incubation. Total RNA was extracted and digested with DNase I (Invitrogen) to remove endogenous genomic DNA and recombinant plasmids. RNA was analyzed by RT-PCR using primers STUB1_cEx2F and STUB1_cEx4R as mentioned above.

## Results

### Clinical Findings

**FIGURE 1:**
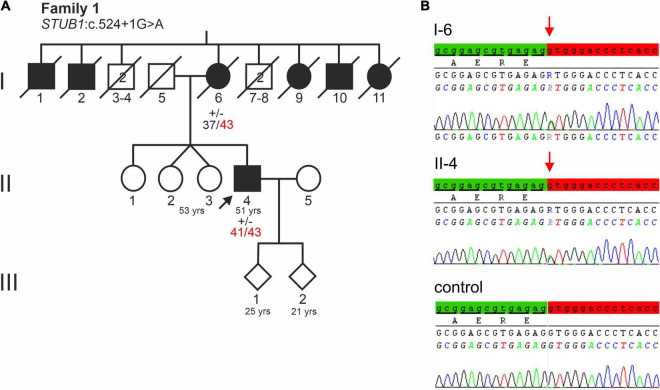
**(A)** Pedigree of a German family with a severe dementia syndrome. The index patient is indicated by an arrow. Black symbols indicate affected family members. Healthy male relatives of generation I are grouped together, their number is indicated in the symbols. Repeat lengths of the *TBP* alleles are indicated. (+/−) indicates heterozygosity for *STUB1* variant c.524+1G>A. **(B)** Electropherograms of *STUB1* sequences of the index patient, his affected mother, and a control. The relevant base change of *STUB1* is indicated. Distal exon 3 sequence is highlighted green, while the intronic sequence is highlighted red.

The male index patient (II-4, Figure 1A) had normal early motor and cognitive development. He finished school after 9th class and then completed a three-year apprenticeship as a locksmith. First signs and symptoms were observed at age 34 years. According to his wife, he had shown word finding difficulties as well as decreased frustration tolerance and social withdrawal, with the latter initially suspicious of depression. Soon after, deficits were noted at his workplace, such as general slowing and clumsiness. He first presented at age 37 years with normal neurological findings, yet accompanied by shallow affect. Cognitive abilities, however, decreased rapidly, leading to early retirement 1 year after initial presentation. At age 39, tests such as Consortium to Establish a Registry for Alzheimer’s Disease (CERAD) battery (which includes Mini-Mental State Examination, Modified Boston Naming Test, verbal fluency, learning of word lists, vocabulary test, construction ability and a test of visuomotor tracking) (Chandler et al., 2005), the Clock-test (Shulman et al., 1993), the Porteus Maze Test (Porteus, 1950), and the Executive Interview (EXIT25) (Royall et al., 1992) revealed a range of cognitive symptoms involving memory, language, praxis, psychomotor speed, attention, and executive functions suggestive of FTD.

Whereas magnetic resonance imaging (MRI) had only shown mild cerebellar atrophy, repeated fluorodeoxyglucose positron emission tomography (PET) scans were largely unremarkable. A dopamine transporter scan using single-photon emission computed tomography was performed to exclude Lewy body dementia (Supplementary Figure 1). Autoimmune or paraneoplastic etiologies were excluded by cerebrospinal fluid (CSF) analysis.

For 6 years, the patient was followed up as an outpatient until he moved into a nursing home at age 44 because of severe disability, including incontinence. At present (age 51), the patient is fully dependent for locomotion and feeding. He is aphasic and agnostic and shows pronounced apraxia as well as generalized spasticity. He exhibits oral motor and vocal tics as well as disinhibited behavior.

The eldest sister of the index patient (II-1, Figure 1A) was initially reported to be healthy but she attended a school for the learning disabled. In her late 40s, she developed personality changes such as inappropriate behavior and aphasia. The index patient also had monozygotic twin sisters (II-2, II-3 of Figure 1A), one of whom has suffered from epilepsy since the age of 18. She was found to have a benign retroorbital tumor that had caused visual disturbances and was surgically removed. The sisters refused genetic testing.

The patient’s deceased mother (I-6, Figure 1A) initially developed memory and behavioral problems in her early 40s. Subsequently, a dementia such as Alzheimer’s disease was assumed, but no precise etiological classification was made. Because of rapid dementia progression, she moved to a nursing home at age 45. She eventually developed spasticity in all four limbs. In consequence, she was severely impaired in all activities of daily living, becoming bedridden before the age of 50. She died at age 79.

Notably, of her nine siblings, five had a dementia syndrome (I-1, I-2, I-9, I-10, I-11 of Figure 1A). Her husband, father of the index patient (I-5, Figure 1A) was not known to have any behavioral or neurological disorder. He died of an unspecified lung condition at age 70. The index patient’s two children, aged 25 and 21, are both healthy.

### Genetic Findings

Genes most frequently associated with early onset dementia, including *PSEN1*, *PSEN2*, *APP*, *MAPT*, *PRNP*, *c9orf72*, were excluded in the index patient and his affected mother (II-4 and I-6 of Figure 1A). Furthermore, repeat length was in the normal range at *C9orf72* (FTD/amyotrophic lateral sclerosis) and *HTT* (HD). Remarkably, repeat expansions in the incomplete penetrance range at locus SCA17 (*TBP*) were detected. The affected mother (I-6, Figure 1A) carried 37/43 CAG/CAA repeats, while her son carried two moderately expanded *TBP* alleles of 41 and 43 CAG/CAA repeats. The expanded alleles showed CAA interruptions in the typical architecture (CAG_3_, CAA_3_, CAG_*x*1_, CAA, CAG, CAA, CAG_*x*2_, CAA, CAG), indicating stable transmission (data not shown).

Sanger sequencing of *STUB1* coding and intronic regions revealed a heterozygous G>A transition at the first base of intron 3 (c.524+1G>A, Figure 1B). The same variant was detected in the affected mother (I-6 of Figure 1A) of the index patient. Analysis tools including MutationTaster2, NetGene2, and ASSP predicted the variant to disrupt the highly conserved splice donor site of intron 3. Interestingly, the wild-type (WT) splice donor of intron 3 was among the three splice sites in *STUB1* with the highest score, i.e., the most conservation (Table 1). In addition to omission of the splice donor by c.524+1G>A, one cryptic splice donor was postulated in exon 3, and four cryptic splice donors and one cryptic splice acceptor were identified in intron 3 by ASSP *in silico* analysis (Table 1).

**TABLE 1:** Localization of *STUB1* wild-type splice sites, alternative/cryptic splice sites in intron 3, and predicted scores.

Localization (intron)	donor/acceptor	Position^1^	flanking sequence	Score^2^
**1**	**donor**	**159**	**CCGCGCGATCgtgagtgcgc**	**6.476**
**1**	**acceptor**	**160**	**tggtccctagACCCGGAACC**	**4.097**
**2**	**donor**	**358**	**CTGCAGCGAGgttggctgac**	**8.622**
**2**	**acceptor**	**359**	**cgttccccagCTTACAGCCT**	**7.226**
*exon 3^3^*	*donor*	*517*	*GCCGCGGAGCgtgagag 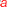 tg*	*4.558*
**3**	**donor**	**524**	**AGCGTGAGAG[g/ 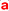 ]tgggaccct**	**10.637**
*3*	*acceptor*	*524* + *94*	*tggcaagcagGAAATGTGG*	*3.235*
*3*	*donor*	*524* + *99*	*AGCAGGAAATgtggggaagt*	*4.739*
*3*	*donor*	*524* + *133*	*TGAGATTGGGgtgtggtcag*	*7.563*
*3*	*donor*	*524* + *138*	*TTGGGGTGTGgtcagacatc*	*4.604*
*3*	*donor*	*524* + *155*	*ATCTGGCCAGgtccatctct*	*6.113*
**3**	**acceptor**	**525**	**caacccccagGGAGCTGGAA**	**5.407**
**4**	**donor**	**612**	**GGCCAAGCACgtgagggtgc**	**5.932**
**4**	**acceptor**	**613**	**ctcttcacagGACAAGTACA**	**8.057**
**5**	**donor**	**669**	**GAAGAGGAAGgtgagtgtgt**	**14.722**
**5**	**acceptor**	**670**	**tgtgccacagAAGCGAGACA**	**5.399**
**6**	**donor**	**786**	**GCACCTGCAGgtgaggcctg**	**17.356**
**6**	**acceptor**	**787**	**gtcactgcagCGTGTGGGTC**	**8.476**

*Localization of wild type splice sites are given in bold. Alternative/cryptic splice sites are given in italic. ^1^First or last cDNA position of the corresponding exon is given. Exonic sequence is given in capitals, intronic sequence in lower letters. Pathogenic variant c.524+1G>A is highlighted in red. ^2^Scores were generated with the Alternative Splice Site Predictor (ASSP). Scores of the preprocessing models reflecting splice site strength, i.e., a position specific score matrix for putative acceptor sites, and a maximum dependence decomposition model for putative donor sites (Wang and Marín, 2006). ^3^This splice donor located in exon 3 was predicted only in the presence of the pathogenic variant c.524+1G>A.*

Variant c.524+1G>A was described only once in 244920 exomes (rs1457745122), resulting in a MAF(A) of 0.000004. No further data or clinical information on the database entry was available. According to ACMG guidelines, variant c.524+1G>A was initially classified as likely pathogenic/class 4; after experimental verification of its pathogenic impact it was reclassified as pathogenic/class 5 (Richards et al., 2015; Abou Tayoun et al., 2018).

To investigate whether an additional pathogenic variant contributed to the severe dementia syndrome of the index patient and his mother, whole exome sequencing was performed on DNA of the index patient. An analysis of all known dementia- and ataxia-related genes revealed no evidence of any further disease-related pathogenic variant.

### Impact of c.524+1G>A on *STUB1* RNA Expression and Processing

**FIGURE 2:**
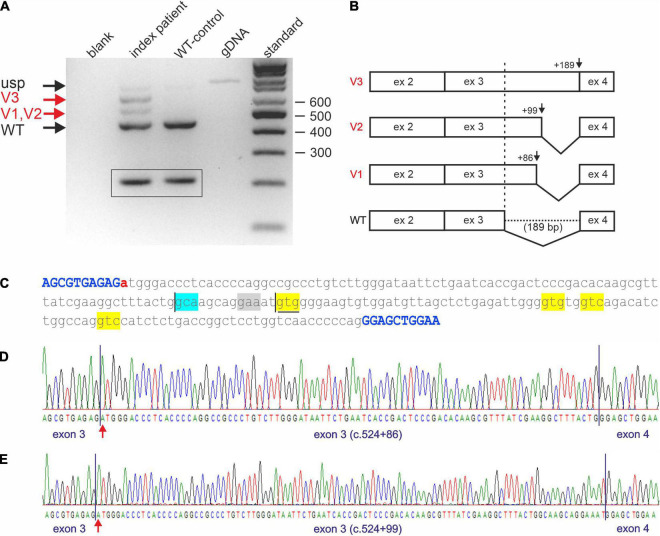
Impaired *STUB1* splicing. **(A)** Expression studies of processed *STUB1* transcripts of the index patient (II-4) and a control. (blank) control without template, (index patient) reverse transcription polymerase chain reaction (RT-PCR) on RNA of the index patient, (WT-control) RT-PCR on RNA of the WT-control, (gDNA) genomic DNA, (standard) 100 bp size standard. Black arrows indicate the correctly processed exon 2–4 fragment of *STUB1* (WT) and the weak, unspliced (usp) product. Red arrows indicate incorrect splicing in the index patient. Splice variants 1, and 2 (V1, V2) correspond to the cryptic splice sites, while splice variant 3 (V3) contains retained intron 3 of *STUB1*. *TBP*-derived 210 bp control fragments for RNA integrity are shown in boxes. **(B)** Graphical overview of impaired splicing (V3-V1) caused by variant c.524+1G>A compared to the correctly spliced wild type (WT) allele. **(C)** Sequence of intron 3 of *STUB1* (lower case) flanked by exons 3 and 4 (upper case, blue). Pathogenic variant c.524+1G>A is highlighted in red. The main cryptic splice site c.524+86 is boxed in blue. Predicted cryptic splice donor sites by Alternative Splice Site Predictor analysis are boxed in yellow, the predicted splice acceptor is boxed in gray. The ultra-rare used cryptic splice site c.524+99 is underlined. **(D)** Electropherogram of products using the cryptic splice site c.524+86. **(E)** Electropherogram of products using the ultra-rare cryptic splice site c.524+99.

Reverse transcription PCR analysis on cDNA of the index patient (II-4 of Figure 1A) and comparison with a cDNA generated from WT whole RNA revealed impaired splicing in the index patient (Figure 2A). While skipping of exon 3 was excluded in the patient, at least three larger fragments were detected (Figure 2A, lane 2) that were not present in the correctly spliced WT control (Figure 2A, lane 3). To verify these additional bands, they were excised from the gel and sequenced directly. Fragments are, from top to bottom (Figure 2A, lane 2; Figure 2B): (a) the unspliced (usp) fragment, (b) without intron 2 but with persisting intron 3 (V3), and (c) without intron 2 but use of a non-predicted cryptic splice donor at position c.524+86 (V1) in intron 3 (Figures 2B–D). The 443 bp fragment (WT, Figure 2A, lane 2), which corresponds to the fragment detected in the WT (Figure 2A, lane 3), is the correctly spliced product of exon 2, exon 3, and exon 4. In the index patient, it was transcribed and processed from his WT allele (Figure 2A, lane 2).

Due to the small size differences in the aberrant spliced fragments of the index patient, direct sequencing was challenging. To avoid missing additional utilized cryptic splice sites, fragments were cloned and multiple clones were re-sequenced. This essentially confirmed the results of the direct sequencing approach. However, one out of 100 clones analyzed used an ultra-rare splice site at position c.524+99 (V2), which was predicted by ASSP analysis (Figures 2C,E and Table 1).

Impaired splicing by variant c.524+1G>A had two main consequences. First, complete retention of intron 3 in the transcripts did not lead to a frameshift, but rather addition of 189 bp of in-frame coding sequence. This corresponds to an insertion of 63 amino acids (p.Arg175_Glu176ins63), which disrupted the coiled-coil domain located at aa 100–127 and aa 170–190 essential for dimerization of CHIP. The same applies to the ultra-rare cryptic splice site at position c.524+99 (p.Arg175_Glu176ins33), which led to the in-frame insertion of an additional 33 aa (Figure 2C). Second, the use of the cryptic splice site at position c.524+86 in intron 3 caused a frameshift (p.Arg175fs*93), resulting in a truncated protein and/or degradation of aberrant RNA *via* nonsense mediated decay.

### *STUB1* Minigene Splicing Studies

To rule out the possibility that the patient’s RNA was degraded and the impaired splicing described above was artificial, two *STUB1* minigenes containing the genomic sequence of parts of intron 1 to intron 4 were constructed: pcDNA3.1-*STUB1*-WT (corresponding to the wild-type sequence) and pcDNA3.1-*STUB1*-Mut (carrying the variant c.524+1G>A). CHO cells were transiently transfected with one of each. CHO cells were selected used since the homology of the coding regions of *STUB1* between *Cricetulus griseus* and *Homo sapiens* is only 89%, allowing reliable assignment of the detected fragments.

**FIGURE 3:**
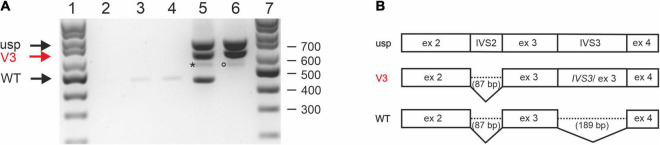
Splicing analysis using *STUB1* minigene constructs. **(A)** Expression studies of *STUB1* minigene templates. (1) 100 bp size standard, (2) control without cDNA, (3) Chinese hamster ovary (CHO) cells mock transfected, (4) CHO cells transfected with vector pcDNA3.1+, (5) CHO cells transfected with construct pcDNA3.1-*STUB1*-WT, (6) CHO cells transfected with construct pcDNA3.1-*STUB1*-Mut, (7) 100 bp size standard. Black arrows indicate wild type (WT) and unspliced (usp) fragments. Retention of intron 3 is marked by a red arrow (V3; also in panel 3B). An asterisk indicates a 530 bp splice intermediate lacking intron 3 but containing intron 2 (lane 5). The rare use of cryptic splice site c.524+86 is marked by a circle (lane 6). **(B)** Graphical overview of detected splice products.

Whole RNA was extracted and analyzed by RT-PCR (Figure 3A). RT-PCR on whole RNA derived from cells transfected with pcDNA3.1-*STUB1*-WT (Figure 3A, lane 5) revealed fragments that correspond (a) to an unspliced (usp) fragment, (b) a splice-intermediate fragment without intron 2 (V3 of Figures 3A,B), and (c) a WT-fragment of 443 bp (WT of Figure 3A, lane 5; Figure 3B). Sequencing confirmed that it was the correctly spliced human mRNA (exon 2, exon 3, and exon 4). An additional faint fragment of 530 bp was detected, which corresponds to a splice intermediate lacking intron 3 but containing intron 2 (Figure 3A, lane 5, asterisk).

In contrast, RT-PCR on RNA extracted from pcDNA3.1-*STUB1*-Mut transfected cells (Figure 3A, lane 6) revealed the fragment containing intron 3 (V3) as was seen in the index patient’s RNA (Figure 2A). The use of the cryptic splice site c.524+86 was barely detectable in the minigene assay. The corresponding 529 bp fragment was only rarely identified (Figure 3A, lane 6, circle). However, no WT fragment of 443 bp was detected in pcDNA3.1-*STUB1*-Mut cells. Furthermore, no 277 bp fragment was detected that would result from skipping of exon 3 (Figure 3A, lane 6).

## Discussion

We report on a family with a severe dementia syndrome caused by impaired splicing of *STUB1* that resulted in retention of intron 3. Repeat expansion of *TBP* alleles in the incomplete penetrance range may contribute to the phenotype observed.

Prominent symptoms of the two family members studied included rapidly progressive cognitive decline in combination with personality change and complete loss of executive function. Clinically, we assumed an FTD syndrome but failed to find typical paraclinical (MRI, PET, CSF) evidence. In SCA48 patients, cognitive impairment may occur years before the movement disorder appears (Genis et al., 2018; De Michele et al., 2019; Palvadeau et al., 2020; Roux et al., 2020; Pakdaman et al., 2021; Ravel et al., 2021). In the two patients presented here, the cognitive and executive decline was, however, so pronounced that the possibility of an ataxia syndrome was not raised. Interestingly in a previous collective of 115 FTD patients, pathogenic variants in *STUB1* were not identified (Roux et al., 2020).

We identified the pathogenic variant c.524+1G>A, which affects the highly conserved splice donor located in intron 3 of *STUB1*, as the underlying disease mechanism in the family described here. To date, more than 90 different pathogenic variants in *STUB1* have been associated with SCAR16 and/or SCA48 (Magri et al., 2022). However, including c.524+1G>A, only eight variants are known to affect conserved splice boundaries in *STUB1*, some of which were detected independently in unrelated families (Cordoba et al., 2014; Olszewska and Kinsella, 2020; Roux et al., 2020; Saft et al., 2021; Magri et al., 2022). Among these, variant c.524+1G>A is the only *STUB1* splice site variant for which impaired splicing has been experimentally demonstrated. Thus, it could be shown that retention of intron 3 predominantly occurred, while two different cryptic splice donors were activated in intron 3 to a lesser extent. Of the two cryptic splice sites used, only the extremely rarely used splice site c.524+99 was predicted by ASSP *in silico* analysis; the mainly used cryptic splice site c.524+86 was only detected experimentally. As suggested (Abou Tayoun et al., 2018), splice site variants should be classified as pathogenic only after experimental evidence of aberrant splicing.

Postulated consequences of the intron 3 retention caused by c.524+1G>A include insertion of an additional 63 aa (p.Arg175_Glu176ins63) in the reading frame of CHIP, which causes loss of the coiled-coil domain and limits the protein’s dimerization capacity. Another possibility of intron 3 retention is gain of function from incorporation of an additional 63 aa. The same mechanism applies to the ultra-rare splice variant c.524+99, which leads to an 33 additional aa (p.Arg175_Glu176ins33) that also disrupts the coiled-coil domain. In contrast, the use of cryptic splice site c.524+86 will result in a frameshift (p.Arg175fs*93) that will abolish the function of the C-terminal E3 ubiquitin ligase. A reduction or loss of function of CHIP due to impairment of either the N-terminal TRPs or the C-terminal ubiquitin ligase has already been discussed (De Michele and Santorelli, 2021). However, some groups have reported a toxic gain of function for missense or frameshift variants of *STUB1* (Genis et al., 2018; De Michele et al., 2019; Chen et al., 2020). A possible gain of function due to incorporation of additional amino acids has not been previously postulated in CHIP pathology.

If a conserved splice site has lost its function due to a base change, either skipping of an exon or activation of cryptic splice sites is observed in most cases (Wang and Burge, 2008; Scotti and Swanson, 2016). Strikingly, we demonstrated retention of intron 3 as the main consequence of aberrant splicing.

Until recently, transcripts with retained introns were considered non-functional, as they usually lead to a premature stop and are degraded *via* nonsense mediated decay (Wong et al., 2016). However, intron retention has been increasingly described as a mechanism that generates a variety of transcripts and plays a role in both normal cell physiology and disease (Monteuuis et al., 2019). Splicing defects, either caused by aberrant *cis-*acting signals or *trans-*acting regulatory splicing factors, have been linked to the development of dementia syndromes, such as Alzheimer’s disease (Biamonti et al., 2021; Rybak-Wolf and Plass, 2021). Remarkably, partial or complete intron retention has already been described in other genes associated with dementia (Braggin et al., 2019; Li et al., 2021).

By excluding other genes that have been associated with cognitive decline, we observed repeat expansions in the range of reduced penetrance at the SCA17 locus. SCA17 is caused by expansions of a CAG/CAA repeat of *TBP* that results in pathogenic elongation of a polyglutamine (polyQ) stretch. Full penetrance is associated with 49 or more CAG/CAA repeats, while reduced penetrance is observed when the number of CAG/CAA repeats ranges from 41 to 48. Alleles of 41 to 44 repeats have an estimated penetrance of only 50% (Toyoshima et al., 2005/2019).

Here, we detected 43 CAG/CAA repeats in the affected mother of the index patient but compound heterozygous expansions of 41 and 43 CAG/CAA repeats in her affected son. Unfortunately, no DNA samples were available from other family members. Since the 43 CAG/CAA *TBP* allele was stably inherited from the mother, it can be assumed that the 41 allele originates from the father. However, he showed no signs of cognitive decline or movement disorder before his death at age 70. Unstable transmission of a large normal allele seems unlikely, as CAA interruptions typical of stable transmission were detected (Zühlke et al., 2001; Maltecca et al., 2003). Homozygous and compound heterozygous carriers of *TBP* expansions have been described and do not appear to develop disease earlier or to a more severe degree than heterozygous carriers with comparably sized repeats (Zühlke et al., 2003; Toyoshima et al., 2004; Hire et al., 2011). Even in other polyQ disorders, such as HD, carriers of two expanded alleles do not show an earlier onset of disease but may have more rapid disease progression (Lee et al., 2012). Therefore, the two expanded *TBP* alleles observed in the index patient may function as a pacemaker of disease progression.

We can only speculate on the genotypes of the sisters of the index patient. While two sisters are healthy, the oldest sister did not develop personality changes until her late 40s. This could be associated with carrying one of the moderately expanded *TBP* alleles found in the family. Personality changes and psychiatric symptoms have been described in patients who have between 41 and 44 CAG/CAA repeats in *TBP* (Stevanin et al., 2003; Toyoshima et al., 2004; Nolte et al., 2010).

During the preparation of this manuscript, another group independently reported the association of the pathogenic variant c.524+1G>A of *STUB1* in a patient with an extended *TBP* repeat of 42 (Magri et al., 2022). The 56-year-old patient described appeared to be severely affected, as indicated by a Scale for the assessment and rating of ataxia (SARA) score of 40, but his age at disease onset was not reported (Magri et al., 2022). His first symptoms were an ataxic movement disorder, which could not be observed in the family described here due to the devastating course of the disease.

Therefore, the severe impairment in their patient and our index patient was confirmed to be caused by both the pathogenic variant in *STUB1*, which causes intron retention and thus gain of 63 aa in CHIP, and an elongated polyQ segment of TBP. It can be speculated that the two extended *TBP* repeats in the patient presented here exacerbate the phenotype toward an FTD syndrome (Olszewska et al., 2019).

CHIP has already been linked to various neurodegenerative diseases, such as polyQ disorders, Parkinson’s disease, and Alzheimer’s disease (Miller et al., 2005; Kumar et al., 2012; Momtaz et al., 2020; Mylvaganam et al., 2021; Potjewyd and Axtman, 2021). In a transgenic SCA3 mouse model, it has been shown that reduction in CHIP leads to an increase in ataxin-3 microaggregates (Williams et al., 2009). In SCA17, both impairment of TBP binding to polymerase II promoters and formation of toxic polyQ aggregates have already been demonstrated (Friedman et al., 2008). Therefore, it is likely that CHIP dysfunction further enhances this process.

It was recently shown, that inheritance of pathogenic *STUB1* variants in combination with *TBP* alleles of reduced penetrance is frequently of digenic-caused disease with mutant CHIP and TBP has not been definitively elucidated, *STUB1* and *TBP* should be included in diagnostic genetic testing panels for patients with early onset dementia syndromes that resemble FTD.

## Data Availability Statement

The original contributions presented in the study are included in the article/Supplementary Material, further inquiries can be directed to the corresponding author.

## Ethics Statement

The studies involving human participants were reviewed and approved by the Ethics committee of the Justus-Liebig-University of Giessen (AZ24/14erw). The patients/participants provided their written informed consent to participate in this study.

## Author Contributions

DN designed the study. MR, JP, PW, and DN performed laboratory work and analyzed and interpreted the data. AD had initially seen the patients. NA was responsible for the follow-up of patients and family members. DN wrote the first draft of the manuscript and prepared the figures. MS prepared Supplementary Figure 1. MR wrote sections of the manuscript and prepared the tables. MR, JP, NA, PW, AD, and DN contributed to manuscript revision. All authors approved the submitted version.

## Conflict of Interest

The authors declare that the research was conducted in the absence of any commercial or financial relationships that could be construed as a potential conflict of interest.

## Publisher’s Note

All claims expressed in this article are solely those of the authors and do not necessarily represent those of their affiliated organizations, or those of the publisher, the editors and the reviewers. Any product that may be evaluated in this article, or claim that may be made by its manufacturer, is not guaranteed or endorsed by the publisher.
