# Supplementary material for: A Severe Dementia Syndrome Caused by Intron Retention and Cryptic Splice Site Activation in STUB1 and Exacerbated by TBP Repeat Expansions
Source: Front Mol Neurosci. 2022 Apr 14;15:878236. doi: 10.3389/fnmol.2022.878236 (PMC9048483; doi:10.3389/fnmol.2022.878236)
Supplement: Supplementary file 1 [file Image_1.pdf]

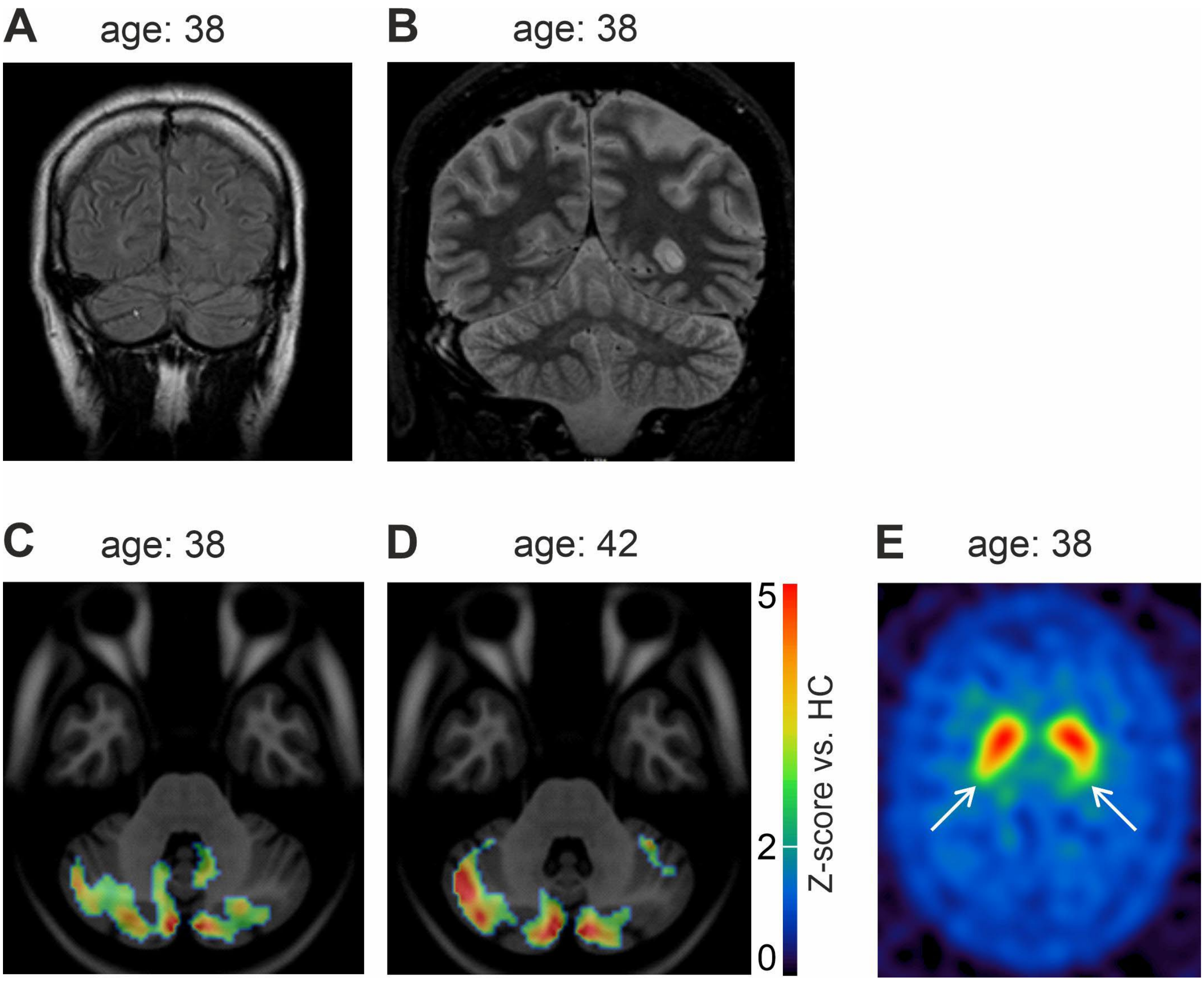


**Supplemental Figure 1.** Brain MRI, PET scan, and DaTscan of index patient II-4. Coronal T1 (A) and T2 (B) sequence show only moderate atrophy of the cerebellum. PET scans at age 38 (C), and age 42 (D) are given. Over the course of four years, the cerebellar glucose hypometabolism of the index patient increases slightly compared to the healthy controls. A DaTscan (E) was performed to exclude Lewy-body-dementia. Striatum shows an inconspicuous distribution pattern of the tracer. White arrows indicate the presynaptic dopamine transporter concentration of the striatum.
