# Supplementary material for: A Severe Dementia Syndrome Caused by Intron Retention and Cryptic Splice Site Activation in STUB1 and Exacerbated by TBP Repeat Expansions
Source: Front Mol Neurosci. 2022 Apr 14;15:878236. doi: 10.3389/fnmol.2022.878236 (PMC9048483; doi:10.3389/fnmol.2022.878236)
Supplement: Supplementary file 2 [file Table_1.DOCX]

**Supplemental Table 1**: Primer used for **(A)** repeat length analysis, **(B)** amplification and sequencing of *STUB1*

| **primer** | **sequence** | **application** |
| --- | --- | --- |
| **A** |  | repeat length analysis |
| HD1_FAM6 | 5-'FAM6-ATGAAGGCCTTCGAGTCCCTCAAGTCCTTC-3' | HD, *HTT* |
| HD3 | 5'-GGCGGTGGCGGCTGTTGCTGCTGCTGCTGC-3' |  |
| C9orf72_F3 | 5'-FAM-AGCAAGCTCTGGAACTCAGGAGTCG-3' | FTD, *C9orf72* |
| C9orf72_R6 | 5'-CCTCACTCACCCACTCGCCAC-3' |  |
| SCA17F_FAM6 | 5'-FAM6-CCTTATGGCACTGGACTGAC-3' | SCA17, *TBP* |
| SCA17R | 5'-GTTCCCTGTGTTGCCTGCTG-3' |  |
| **B** |  | sequencing  *STUB1* |
| Ex1F | 5'-GCCCAGTGTCCCCGTCCAG-3' | exon 1 |
| Ex1R | 5'-CGAAGAGGAGCCAGACCCT-3' | exon 1 |
| Ex2F | 5'-GTACTCCACTGTGCACAGATCC-3' | exon 2 - 3 |
| Ex3R | 5'-GCACTCTTCCAGCTCCCTG-3' | exon 2 - 3 |
| Ex4F | 5'-ACTCCCGACACAAGCGTTTATC-3' | exon 4 - 6 |
| Ex4Fint | 5'-AGGGAGCTGGAAGAGTGC-3' | internal sequencing primer |
| Ex6Rint | 5'-CTGCAGGTGCTCCTCGATG-3' | internal sequencing primer |
| Ex6R | 5'-TCAAAATGACCCACACGCTGC-3' | exon 4 - 6 |
| Ex7F | 5'-ACATCGAGGAGCACCTGCAGG-3' | exon 7 |
| Ex7Fnc | 5'-CTGTTGGACTCTGGACTGTTTC-3' | internal sequencing primer |
| Ex7R | 5'-GGCCCGCAGCTAATCTCTG-3' | exon 7 |
